# Impact of specimen adequacy on the assessment of renal allograft biopsy specimens

**DOI:** 10.1590/1414-431X20165301

**Published:** 2016-04-26

**Authors:** S. Cimen, L. Geldenhuys, S. Guler, A. Imamoglu, M. Molinari

**Affiliations:** 1 Department of Surgery, Dalhousie University, Halifax, NS, Canada; 2 Department of Pathology, Dalhousie University, Halifax, NS, Canada; 3 Department of Urology, Yildirim Beyazit EAH, Ankara, Turkey

**Keywords:** Renal allograft, Biopsy, Specimen adequacy, Banff criteria

## Abstract

The Banff classification was introduced to achieve uniformity in the assessment of renal allograft biopsies. The primary aim of this study was to evaluate the impact of specimen adequacy on the Banff classification. All renal allograft biopsies obtained between July 2010 and June 2012 for suspicion of acute rejection were included. Pre-biopsy clinical data on suspected diagnosis and time from renal transplantation were provided to a nephropathologist who was blinded to the original pathological report. Second pathological readings were compared with the original to assess agreement stratified by specimen adequacy. Cohen's kappa test and Fisher's exact test were used for statistical analyses. Forty-nine specimens were reviewed. Among these specimens, 81.6% were classified as adequate, 6.12% as minimal, and 12.24% as unsatisfactory. The agreement analysis among the first and second readings revealed a kappa value of 0.97. Full agreement between readings was found in 75% of the adequate specimens, 66.7 and 50% for minimal and unsatisfactory specimens, respectively. There was no agreement between readings in 5% of the adequate specimens and 16.7% of the unsatisfactory specimens. For the entire sample full agreement was found in 71.4%, partial agreement in 20.4% and no agreement in 8.2% of the specimens. Statistical analysis using Fisher's exact test yielded a P value above 0.25 showing that - probably due to small sample size - the results were not statistically significant. Specimen adequacy may be a determinant of a diagnostic agreement in renal allograft specimen assessment. While additional studies including larger case numbers are required to further delineate the impact of specimen adequacy on the reliability of histopathological assessments, specimen quality must be considered during clinical decision making while dealing with biopsy reports based on minimal or unsatisfactory specimens.

## Introduction

Rejection continues to be a significant problem following kidney transplantation (1). Renal allograft biopsy for histopathological examination is the technique of choice for diagnosis of rejection (2). In the past, biopsy specimens of transplanted kidneys were interpreted subjectively by pathologists. In 1993, the Banff classification of renal transplant pathology was introduced in order to achieve uniformity in histopathological assessment of renal graft biopsies (3). It has been revised regularly since then by consensus meetings held in the light of cumulative practical experience and feedback (4,5).

Banff classification identifies the changes, which may be seen in dysfunctional renal allografts, defines a grading system for these changes, and places the results into a numeric formulation (6). The goal of this classification is composing a schema, in which a given biopsy grading would imply in a prognosis for the graft function or in a therapeutic response that may influence the choice of therapy (1,7).

Nevertheless, application of the Banff schema can be complex in practice, and observer agreement for rejection diagnosis and grading can vary significantly (8,9). It has already been demonstrated that application of the Banff criteria shows a substantial interobserver variation (8-10). Specimen adequacy assessment is the initial step during the assessment of a renal graft biopsy specimen (1). However, studies that have evaluated interobserver variation using the Banff criteria did not analyze the impact of specimen adequacy (adequate, minimal and unsatisfactory), i.e., the reliability of the reported diagnosis (8 - 10).

Considering that histopathological analysis of renal allograft biopsies based on the Banff classification has crucial clinical implications, the aim of this study was to analyze the impact of specimen adequacy on the diagnostic agreement during application of the Banff criteria at our transplant center. To the best of our knowledge, there are no previous studies in the literature regarding the influence of specimen adequacy (i.e., quality of renal allograft biopsy) on the diagnostic agreement based on Banff criteria.

## Material and Methods

This was a single-center retrospective study approved by Nova Scotia Health Authority Ethical Review Board (File#1015131). Kidney transplant database was reviewed to identify patients who underwent renal allograft biopsy at our center between July 2010 and June 2012. Renal allograft biopsy specimens taken from adult (age >18 years), female/male kidney transplant recipients with the clinical indication of ‘suspicion of acute rejection' were included. Biopsies taken with other clinical indications, protocol biopsies and inconclusive biopsies were excluded. Pre-biopsy clinical data including cause of end stage renal disease, donor type, Human Leukocyte Antigen mismatch status, panel reactive antibody level, date of kidney transplant surgery, date of graft biopsy, immunosuppressive treatment, serum calcineurin inhibitor level, serum creatinine level and indication of graft biopsy were retrieved from patient charts. A transplant nephropathologist, who was not involved in the initial review of these specimens and was blinded to the initial pathology reports, was provided with these clinical data and asked to review the same graft biopsy specimens to report a diagnosis including diagnostic category and grade (when relevant).

The Banff 97 criteria were used during both readings (6,11). Specimens were considered as ‘adequate' when they had at least 10 glomeruli and 2 arteries, and ‘minimal' when they had 7 to 9 glomeruli with 1 artery. Specimens of less quality were deemed ‘unsatisfactory'. The diagnoses of the reviewing nephropathologist were compared with the original diagnoses for interobserver agreement analysis. Identical assessments were categorized as ‘full agreement' (i.e. correlation), presence of minor differences including one degree of difference (i.e., 1 grade difference) in the same category of the classification system was considered as ‘partial agreement' and presence of a higher degree of difference (such as diagnoses in different categories) was deemed as ‘no agreement' (i.e., no correlation).

Cohen's kappa test was used for interobserver agreement analysis. Fisher's exact test was used to compare the full agreement rates among different ‘specimen adequacy' categories. All statistical analyses were carried out with Stata Statistical Software (Release 13, StataCorp LP, USA). The level of significance was set at P<0.05.

## Results

**Figure 1:**
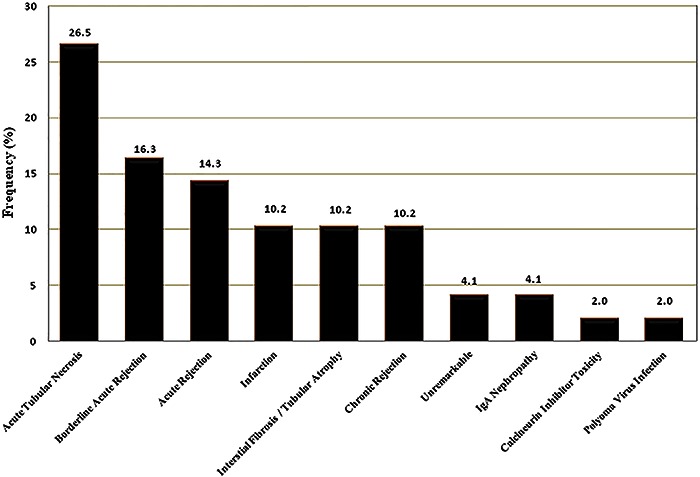
Frequency (%) of histopathological diagnoses from renal allograft biopsy specimens reported by the nephropathologist. IgA, immunoglobulin A.

**Figure 2:**
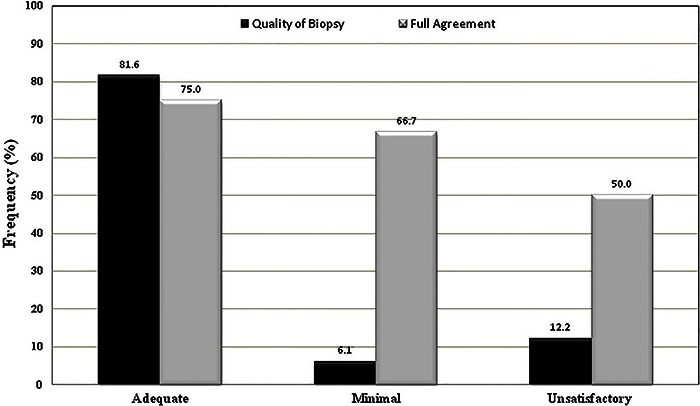
Percentage of full agreement in biopsy specimens stratified by specimen adequacy (quality of biopsy).

A total of 49 graft biopsies were reviewed by the nephropathologist (Figure 1). Among these, 40 (81.6%) were classified as ‘adequate', while 3 (6.12%) and 6 (12.24%) were found ‘minimal' and ‘unsatisfactory', respectively. The agreement analysis among the first and second diagnoses revealed a kappa value of 0.97. There was ‘full agreement' in 75% (30 out of 40) of the adequate specimens between the first and the second readings (Figure 2). On the other hand, ‘full agreement' was found in 66.7% (2 among 3) and 50% (3 among 6) of the ‘minimal' and ‘unsatisfactory' specimens, respectively (Figure 2). There was ‘no agreement' between the two readings in 5% (2 out of 40) of the adequate specimens; this figure went up to 16.7% (1 out of 6) in the assessment of unsatisfactory specimens.

There was ‘full', ‘partial' and ‘no' agreement in 71.4, 20.4, and 8.2% of the entire cohort, respectively. A similar trend was found when these groups were compared in terms of specimen adequacy: 85.7% of the specimens which led to full agreement were classified as ‘adequate' specimens while this figure was 50% in the ‘no agreement' group. Approximately 8% of the full agreement group specimens were classified as ‘unsatisfactory specimens', while they constituted 25% of the ‘no agreement' group specimens.

‘Minimal' and ‘unsatisfactory' specimens and “partial” and “no agreement” categories were combined due to low case numbers for statistical analysis using Fisher's exact test. This test yielded a P value above 0.25 showing that the difference was not statistically significant.

## Discussion

Studies investigating the reproducibility of the Banff criteria started soon after it was first introduced. The preliminary investigations done by Solez et al. (12) and Marcussen et al. (13), which were based on the 1991 Banff classification concluded that the Banff criteria was reproducible. However, the researchers were involved in the development of these criteria and they analyzed only the adequate biopsy specimens (3).

Furness et al. worked on cases in which doubt had been expressed regarding the diagnosis of acute rejection (8). In these cases definitive diagnoses were confirmed from the subsequent clinical course. In another study, post-transplant clinical data was deliberately withheld from the pathologists, whom were asked to either ‘confirm or exclude' acute rejection referring to the Banff 1991 classification (3). They found that interobserver agreement level was low. Notably, these authors did not include the minimal or unsatisfactory specimens in their study.

Furness and Taub evaluated interobserver agreement of the Banff classification (updated in 1997) in terms of both acute and chronic histopathological changes (9). They concluded that interobserver agreement was alarmingly low. However, it must be acknowledged that pathologists were blind to the pre-biopsy clinical information, which could have influenced their interpretations and increased interobserver agreement.

In another investigation based on the Banff classification's 1997 update, Gough et al. (14) assessed protocol biopsies. They evaluated the degree of interobserver variation between two pathologists for the diagnoses of ‘no acute rejection', ‘borderline rejection' and ‘acute rejection'. They reported good agreement in diagnosing acute rejection. Nevertheless, these researchers excluded the minimal or unsatisfactory specimens.

Veronese et al. (10) worked with protocol biopsies as well. They evaluated interobserver variation referring to the Banff 1997 classification (11). This investigation concluded that, while there was good interobserver agreement in diagnosing acute rejection, grading the acute rejection displayed substantial interobserver variation. However, the pathologists were blind to pre-biopsy clinical data of the patients and inadequate specimens were not included in this study.

In all these previous studies, clinical data of the patients were intentionally withheld from the pathologists since they focused mainly on the feasibility and reproducibility of this classification. In addition, they often included protocol biopsies rather than biopsies performed based on indication, such as a clinical suspicion of acute rejection. Since these studies did not include minimal or unsatisfactory specimens, it was not possible to evaluate the impact of specimen adequacy on observer agreement.

In our study, we focused on the diagnostic agreement in a clinical setting during the interpretation of renal allograft specimens taken when clinically indicated. In line with this approach, the nephropathologist was not blind to the indication of biopsy and to the pre-biopsy clinical data. Given that it is not unusual for a nephropathologist to review a suboptimal biopsy specimen in clinical settings, we did not exclude those specimens. We sought for their impact on diagnostic agreement and we found - though not statistically significant - a ‘clinically significant' lower agreement rate when assessing minimal or unsatisfactory specimens.

Despite the fact that pathologist's comments regarding the specimen adequacy (adequate/minimal/unsatisfactory) are always given in the graft biopsy reports, our observation is that this information is usually underappreciated by clinicians probably due to the lack of scientific data regarding the reliability of minimally satisfactory or unsatisfactory specimens. In our study, we found a higher diagnostic discrepancy among pathologists during the interpretation of less than adequate specimens.

Thus, our findings indicated that specimen adequacy may be a determinant of diagnostic agreement in the histopathological assessment of renal allograft specimens taken due to clinical suspicion of acute rejection. While additional studies are required to confirm the statistical significance, specimen adequacy must be kept in mind during clinical decision making, especially while dealing with graft biopsies reported as minimal or unsatisfactory specimens.
